# The Case for Intermittent Carbapenem Dosing in Stable Haemodialysis Patients

**DOI:** 10.3390/antibiotics9110815

**Published:** 2020-11-16

**Authors:** Vanda Ho, Felecia Tay, Jia En Wu, Lionel Lum, Paul Tambyah

**Affiliations:** 1 Department of Medicine, National University Hospital, National University Health System, Singapore 119228, Singapore; lionel_lum@nuhs.edu.sg (L.L.); paul_anantharajah_tambyah@nuhs.edu.sg (P.T.); 2 Yong Loo Lin School of Medicine, National University of Singapore, Singapore 119077, Singapore; felecia.tay@mohh.com.sg; 3 Department of Pharmacy, National University Health System, Singapore 119228, Singapore; jia_en_wu@nuhs.edu.sg

**Keywords:** intermittent dosing, meropenem, extended spectrum β-lactamase, haemodialysis, drug monitoring

## Abstract

Purpose: Antimicrobial resistant infections are common in patients on haemodialysis, often needing long courses of carbapenems. This results in a longer hospital stay and risk of iatrogenic complications. However, carbapenems can be given intermittently to allow for earlier discharge. We aim to describe the clinical outcomes of intermittent versus daily meropenem in stable, intermittently haemodialysed patients. Methods: In total, 103 records were examined retrospectively. Data collected include demographics, clinical interventions and outcomes such as hospital length of stay (LOS), 30-day readmission rates and adverse events. Findings: Mean age 61.6 ± 14.2 years, 57.3% male. Most common bacteria cultured were Klebsiella pneumoniae (16.5%). The most common indication was pneumonia (27.2%). Mean duration of therapy on meropenem was 12.4 ± 14.4 days; eight patients needed more than 30 days of meropenem. In total, 55.3% did not have intervention for source control; 86.4% received daily dosing of meropenem; 7.8% patients received intermittent dosing of meropenem only, and 5.8 patients received both types of dosing regimens. LOS of the index admission was shorter for the intermittent arm (15.5 ± 7.6 days versus daily: 30.2 ± 24.5 days), though 30-day readmission was higher (50% versus daily: 38.2%). Implications: We recommend further rigorous randomised controlled trials to investigate the clinical utility of intermittent meropenem dosing in patients on stable haemodialysis.

## 1. Background

End-stage renal failure (ESRF) patients on haemodialysis often have multiple co-morbidities and are susceptible to bacterial infections [1] due to a variety of reasons related to the host [2] and surroundings [3]. In many parts of the world, these infections are often caused by extended spectrum β-lactamase (ESBL)-producing Gram-negative bacteria requiring antibiotics such as meropenem, which can be only administered intravenously [4].

Renally adjusted meropenem in haemodialysis patients is administered once daily based on product labelling. Infections such as osteomyelitis or pyogenic abscesses require several weeks of treatment with antibiotics. This often results in prolonged hospitalisation, an admission to subacute hospital to complete the antibiotic course or the use of outpatient antibiotic therapy services. ESRF patients would receive meropenem post-dialysis on haemodialysis days and come to the hospital for administration of meropenem on non-haemodialysis days. However, intravenous access is required. As most patients on haemodialysis often present with poor venous access, for long-term antibiotics, usually, a peripherally inserted central catheter (PICC) would be used. This would also logistically be easier by reducing the need to re-site intravenous cannulas every 2 to 3 days. This may be a problem in haemodialysis patients who commonly have limited vascular access and are more susceptible to line-related sepsis. Furthermore, there is significant stress from logistics on the patient and their caregiver. 

An alternative meropenem regime involving intermittent thrice-weekly dosing administered post-dialysis may be feasible. There are reports demonstrating its efficacy [5]. Pharmacokinetic data suggest that reduced clearance in ESRF patients leads to longer half-life of meropenem and this may be sufficient to achieve effective concentrations with intermittent dosing [6]. Practically, there are many potential benefits. This regime may reduce hospital length of stay (LOS) and the need for PICC, thus reducing its associated costs and complications, such as nosocomial infections. It also circumvents the logistical issues of daily administration at a healthcare institution. This would reduce healthcare utilisation and improve health-related quality of life. 

The evidence for toxicity with high-dose intermittent meropenem is not clear [7,8], though meropenem and its metabolite have also been shown to be effectively removed by haemodialysis [6]. Unfortunately, intermittent meropenem dosing regimens for haemodialysis patients have not been widely used due to limited published data. 

To investigate the feasibility of an intermittent meropenem dosing regime in ESRF patients, we describe clinical outcomes of intermittent versus daily meropenem in stable, intermittent haemodialysed patients in terms of length of hospital stay and 30-day readmission rates.

## 2. Materials and Methods

A single-centre retrospective cohort study was conducted in the National University Hospital (NUH), Singapore. Data were obtained from the NUH’s Antimicrobial Stewardship Programme database from May 2015 to February 2017. Inclusion criteria were adult patients (≥21 years old) on stable intermittent haemodialysis who had received at least 3 days of meropenem. Our study was approved by the local ethics board (domain specific review board DSRB approval number: 2018/00578).

In total, 103 patient records were examined. Data collection categories were patient demographics, clinical data, meropenem regime and clinical outcomes, as detailed in Appendix A. Demographic data included age, sex, ethnicity and Charlson Comorbidity Index (CCI) [9]. Involvement of the infectious disease team was documented. Indication for haemodialysis (e.g., diabetic nephropathy) and the haemodialysis regime (i.e., intermittent haemodialysis thrice weekly) were recorded. Patients were considered immunosuppressed if they had an active malignancy, on active chemotherapy, on high dose of steroids or other immunosuppressants. Clinical data included site of infection, microbiological cultures (both poly-microbial and monomicrobial infections), antimicrobial susceptibilities and interventions performed for source control. The aim of administering meropenem (i.e., cure; eradication of infection versus suppression; long-term course of antibiotics to reduce the incidence of clinical flares of the infection), dosing regimen (i.e., intermittent, daily or both) and duration were also included. Meropenem 500 mg and 1000mg given as a bolus; 2000 and 3000 mg were given as a 30 minutes-long infusion. Clinical outcomes included LOS during the index admission, the intensive care unit (ICU) LOS (if any), 30-day readmission rate, number of relapses with the same microorganism within one year, total LOS within the year for the same infection, adverse events specific to meropenem and mortality within one year.

As this study was conducted retrospectively and group sizes are different, descriptive analyses were used. No statistical comparisons were carried out. Results are reported to 1 decimal place.

## 3. Results

In total, 103 patient records were examined.

### 3.1. Demographics

Mean age was 61.6 ± 14.2 years, of which 57.3% were male (*n* = 59). The ethnic distribution was comparable to the Singapore population distribution. Overall, 81.6% were from government subsidised wards (*n* = 89). Moreover, 56.3% were admitted under nephrology (*n* = 58), followed by surgery (*n* = 29, 28.2%), and 41 (39.8%) were referred to the infectious disease team.

Overall, 75.7% were not immunocompromised (*n* = 78). Moreover, 17 (16.5%) had active malignancy, 6 (5.8%) were on chemotherapy and 7 (6.8%) were on long term steroids. 

The majority were on dialysis due to diabetic renal disease (*n* = 77, 74.8%), followed by glomerulonephritis (*n* = 9, 8.7%) and hypertensive renal disease (*n* = 7, 6.8%).

### 3.2. Microbiological Results

Samples obtained were mainly from blood (*n* = 63, 61.2%). Around a quarter (*n* = 34, 33.0%) were from wound swabs, and a minority (*n* = 13, 12.6%) were from urine cultures. Around half (*n* = 53, 51.5%) did not have any positive microbiological result. The most common bacteria cultured were *Klebsiella pneumoniae* (*n* = 17, 16.5%), followed by *Escherichia coli* (*n* = 11, 10.7%), *Staphylococcus aureus* (*n* = 10, 9.7%), *Pseudomonas aeruginosa* (*n* = 9, 8.7%), *Enterobacter cloacae* (*n* = 7, 6.8%) and *Streptococcus pneumoniae* (*n* = 4, 3.9%). When cultured, bacteria were sensitive to meropenem by EUCAST (European Committee on Antimicrobial Susceptibility Testing) criteria. 

### 3.3. Clinical Interventions

The most common diagnosis was pneumonia (*n* = 28, 27.2%), followed by skin and soft tissue infections (*n* = 22, 21.4%).

Meropenem was largely used with curative intent (*n* = 65, 65.4%). In total, 61 patients had bacteria which were resistant to third-generation cephalosporins. Nine patients had bacteria which were reported susceptible to cephalosporins, but clinical decision was made to continue meropenem by the primary treating physicians despite culture result due to patient’s clinical condition. In contrast, meropenem was used for suppression of chronic infections in nine (7.9%) patients. Two were on meropenem due to hypersensitivity to other narrow-spectrum antibiotics. Mean duration of therapy on meropenem was 12.4 ± 14.4 days, with eight patients requiring more than 30 days of meropenem. The longest duration was a total of 109 days. 

The majority received daily dosing of meropenem (*n* = 89, 86.4%). Eight (7.8%) patients received intermittent dosing of meropenem only, and six (5.8%) patients received both types of dosing regimens. All patients on intermittent dosing were on a thrice-weekly dosing regime. Dosing of meropenem varied, with eight (57.1%) patients on 2 g/2 g/3 g dosing, three (21.4%) on 2 g/2 g/2 g, two (14.3%) on 1 g/1 g/1 g and one (7.1%) on 1 g/1 g/2 g. This variation of dosing was also seen in the daily dosing regime, with a range from 500 mg once to twice daily to 1 g once to thrice daily. Comparison of variables amongst different meropenem dosing regimes is presented in Table 1.

In total, 55.3% did not have any intervention for source control (*n* = 57). Of those who received interventions, 8 (7.8%) had percutaneous drainage, 21 (20.4%) underwent major surgery and 15 (14.6%) had minor surgical procedures. 

### 3.4. Clinical Outcomes 

Mean LOS in hospital was 33.7 ± 3.9 days. In total, 29 (28.2%) required ICU admission, with a mean ICU LOS of 2.1 ± 0.6 days. LOS was longer in the group with continuous meropenem (30.2 ± 24.5 days versus 15.5 ± 7.6 days), though antibiotic duration was shorter (9.0 ± 6.3 days versus 23.4 ± 17.5 days). This longer duration of antibiotics despite shorter LOS is possible as patients on intermittent meropenem dosing receive their doses as outpatients at their dialysis centres. 

In total, 93.2% (*n* = 96) did not have any documented adverse events. Four patients developed *Clostridium difficile* colitis, two developed allergic reactions and one acquired carbapenem-resistant *Enterobacteriaceae* (CRE) positive on screening. In total, 41 (39.8%) were readmitted within 30 days. Moreover, 28 (27.2%) had relapses within the year, while 34 (33.0%) died within 1 year of discharge. Of the 20 patients with known cause of death, 7 died from cardiac-related causes, 3 from ESRF and 10 from infection-related causes, mostly secondary to pneumonia. 

## 4. Discussion

To our knowledge, this is the first study to describe clinical outcomes of intermittent versus daily dosing of meropenem in patients on a stable haemodialysis regime. There is evidence that intermittent dosing of beta lactams is safe and effective for severe infections [10]. Whilst intermittent dosing in patients on haemodialysis has been used with other beta lactam antibiotics such as cefepime [11] and cefazolin [12], this approach has not been as widely adopted with carbapenems despite pharmacokinetic and pharmacodynamic data [13,14].

There were few adverse events in patients treated with both intermittent and daily meropenem, in line with previous reports on the tolerability of meropenem [15].

Our data reveal that there is much variation in meropenem dosing for both daily and intermittent dosing. This lack of standardisation highlights the need for interventional trials to clearly determine the optimal dosing regimen. 

In our small cohort, intermittent dosing appears to be a feasible approach to reduce healthcare utilisation, with comparable clinical efficacy to the more widely used daily dosing. The trend where patients on intermittent dosing had reduced hospital stay without major adverse events is encouraging.

There is much impetus to adopt intermittent dosing of meropenem in ESRF patients on haemodialysis. Patients on haemodialysis tend towards developing depression and poor quality of life [16], which have been found to correlate with longer length of stay in hospital [17,18]. An approach which reduces hospital stay and allows the patient to resume normal activities is likely to be welcomed. This is especially so in the era of escalating healthcare costs, which is a major concern for haemodialysis patients [19].

### Strengths and Limitations

We believe that our small study would help to pave the way towards use of intermittent dosing in our haemodialysis population, with the potential to reduce hospital costs and improve the quality of life of our patients.

As this was an exploratory retrospective study, our sample size was small and dosing arms were unequally distributed. We did not have a control cohort. Hence, our study was limited to being descriptive, with no statistical analysis. We were unable to conclude non-inferiority in both approaches or draw conclusions on causality. To increase the sample size, we included a small number of patients on renal replacement therapy secondary to sepsis-induced renal failure. Information on time to resolution of symptoms and clearance blood culture were inconsistently present in our record review and hence were not included in our results. 

## 5. Conclusions

Our study’s findings are preliminary. We recommend for further rigorous randomised controlled trials to investigate the clinical utility of intermittent meropenem dosing in patients on stable haemodialysis. 

## 6. Declarations

Ethics approval and consent to participate: Domain Specific Review Board under National Healthcare Group, reference number 2018/00578

## Figures and Tables

**Table 1:** Comparison of different meropenem dosing regimens.

Characteristics	Daily (*n* = 89, 86.4%)	Intermittent (*n* = 8, 7.8%)	Both (*n* = 6, 5.8%)
Demographics			
Age in years (mean ± SD)	61.8 ± 14.8	57.8 ± 8.0	63.2 ± 12.3
Males (*n*, %)	50 (56.2)	4 (50.0)	5 (83.3)
Charlson Comorbidity Index (mean ± SD)	7.9 ± 2.9	8.4 ± 1.7	8.0 ± 2.2
Immunocompromised (*n*, %)	22 (24.7)	2 (25.0)	1 (16.7)
Haemodialysis indication due to diabetes (*n*, %)	65 (73.0)	7 (87.5)	5 (83.3)
Discipline of admission (*n*, %)			
Renal	54 (60.7)	2 (25.0)	2 (33.3)
Surgery	21 (23.6)	4 (50.0)	4 (66.7)
Referral to infectious disease team (*n*, %)	32 (36.0)	5 (62.5)	4 (66.7)
Clinical Interventions			
Intervention performed for source control (*n*, %)	34 (38.2)	2 (25.0)	0
Purpose of meropenem (*n*, %)			
Cure	56 (63.6)	5 (62.5)	5 (100.0)
Suppression	7 (8.0)	2 (25.0)	0
Empirical	25 (28.4)	1 (12.5)	0
Duration of meropenem, days (mean ± SD)	9.0 ± 6.3	23.4 ± 17.5	47.8 ± 34.6
Clinical outcomes			
LOS of index admission, days (mean ± SD)	30.2 ± 24.5	15.5 ± 7.6	109.7 ± 119.6
ICU length of stay, days (mean ± SD)	2.5 ± 6.1	0	0
Adverse events (*n*, %)	5 (5.6) Clostridium difficile colitis (*n* = 2) Carbapenem-resistant Enterobacteriaceae colonisation (*n* = 1) Drug hypersensitivity (*n* = 2)	0	2 (33.3) *Clostridium difficile* colitis (*n* = 2)
30-day readmissions (*n*, %)			
1	31 (34.8)	4 (50.0)	3 (50.0)
2	3 (3.4)		
Relapses within one year (*n*, %)			
1	14 (16.1)	2 (25.0)	2 (33.3)
2	5 (5.8)	1 (12.5)	0
3	1 (0.9)	1 (12.5)	0
4	0	0	0
5	0	0	0
Mortality within 1 year (*n*, %)	29 (32.6)	3 (37.5)	2 (33.3)

## Appendix A. Study Pro-Forma

Patient No:

### Appendix A.1. Patient Information

**Age (as of D1 Meropenem)**	**Height (cm)**	**Weight (kg)**	**Sex:** **M/F**	**Class:** **A/B1/B2/C**	**Ethnicity:** **Chinese/Malay/Indian/Others (Specify)**
Charlsons Comorbidity Index score: PMH (circle where appropriate): DM, HTN, Hyperlipidemia, IHD, CVA, PVD Any immunocompromised state(s): Chemotherapy, TNF blockers, steroids, DXT, others – please specify: Indication for HD: DM, HTN, ADPKD, GN, AIN, sepsis Others – please specify:			Discipline of admission (circle where appropriate): Renal/RCCM/Cardio/Endocrine/Gastro/Neuro/Rheum/GRM/Gen Med ID referral: Y/N		
			Haemodialysis regime (circle where appropriate): 3x/week 4x/week Daily SLED		

### Appendix A.2. Infection

Anatomical site of infection: Lung/skin and soft tissue/bones and joints/intra-abdominal/urinary tract/blood/lines/nil source	Initial microbiological cultures result and sensitivities: Histopathological results (if any)
Intervention for source control: □ Percutaneous drainage □ Surgery □ N/A Details:	Purpose of meropenem treatment: □ Cure □ Suppression
Meropenem treatment: Start date: Stop date: Total number of days on meropenem: Regime: daily intermittent If intermittent - Frequency: 3x/week others, please specify: - Dosing (g)	

### Appendix A.3. Clinical Outcomes

Adverse events from meropenem (circle where appropriate):

- Seizure/anaphylaxis/allergy

Total length of hospital stay (days):

Number of readmissions in 30 days:

- Reason for admission:

Number of clinical relapses within 1 year:

- If yes, total length of hospital stay for the same infection in 1 year:

Mortality within 1 year: Y/N

- If yes, CCOD:
